# Characterization of *Helicobacter pylori* Outer Membrane Vesicles over time, in biofilm and planktonic phenotypes

**DOI:** 10.3389/fmicb.2026.1765988

**Published:** 2026-04-20

**Authors:** Valentina Puca, Beatrice Marinacci, Sara Pagotto, Paweł Krzyżek, Federica Di Cintio, Benedetta Pellegrini, Laura Pietrangelo, Maurizio Ronci, Rossella Grande

**Affiliations:** 1 Department of Pharmacy, University “G. d’Annunzio” of Chieti-Pescara, Chieti, Italy; 2 Department of Medical, Oral and Biotechnology Sciences, University “G. d’Annunzio” of Chieti-Pescara, Chieti, Italy; 3 Center for Advanced Studies and Technology (CAST), University “G. d’Annunzio” of Chieti-Pescara, Chieti, Italy; 4 Department of Microbiology, Faculty of Medicine, Wroclaw Medical University, Wrocław, Poland; 5 Department of Medicine and Aging Sciences, University “G. d’Annunzio” of Chieti-Pescara, Chieti, Italy

**Keywords:** biofilm, exoproteome, *Helicobacter pylori*, Outer Membrane Vesicles (OMVs), time-course analysis

## Abstract

**Introduction:**

*Helicobacter pylori* is known to be a major pathogen causing gastric diseases via its direct colonization of the gastric mucosa. *H. pylori* releases Outer Membrane Vesicles (OMVs) throughout the growth process both in planktonic and biofilm phenotypes. The number, size and content of *H. pylori* OMVs over time, especially in *H. pylori* biofilm, remain unclear.

**Methods:**

In this study, we analyzed *H. pylori* biofilm at 2, 6, and 10 days as well as we extracted and characterized *H. pylori* pOMVs and bOMVs over time by transmission electron microscopy, nanoparticle tracking analysis, dynamic light scattering, electrophoretic light scattering and proteomic technology.

**Results:**

*Helicobacter pylori* ATCC 43629 formed a multi-structured biofilm with large clusters characterized by mostly live cells and some fractures corresponding to water channels. Analysis of *H. pylori* OMVs reveals that the bacterial growth time and phenotype affect their number, size, and composition. Proteomic analysis revealed that in the early growth phase pOMVs are enriched with multiple virulence factors associated with host cell destruction whereas during later growth phases vesicles contain factors involved in the metabolic processing. The proteome of bOMVs was much more homogeneous and stable over time: in late growth stages, bOMVs proteomic analysis identified proteins involved in iron accumulation, protection against oxidative stress, immunosuppression in the gastric environment, and virulence promoting inflammation and tumorigenesis.

**Conclusion:**

This study suggests that *H. pylori* induces pathogenicity at least partially by secreting bOMVs that could promote tissue destruction related to tumorigenesis; therefore, the development of gastric cancer could be associated not only with the microorganism itself, but also with OMVs that it produces.

## 1 Introduction

*Helicobacter pylori* is a spiral Gram-negative microorganism colonizing the gastric mucosa and it is associated with the development of gastritis, peptic ulcer disease, Mucosa-Associated Lymphoid Tissue lymphoma (MALT) and gastric cancer (Eshraghian, 2014). Although the human host and in particular the gastric environment represents the favorite niche of *H. pylori*, this microorganism has been detected also in the natural environment, outside the host (Percival and Suleman, 2014). The survival and persistence of the infection is allowed by an extraordinary ability to adapt itself to unfavorable environment both trough the biofilm formation and the modification from spiral to coccoid morphology (Krzyżek and Grande, 2020; Krzyżek et al., 2020). *H. pylori* is capable to adopt several survival strategies among which the capability of developing biofilm, a complex structure characterized by cells adhered to a biotic or abiotic surface and protected by a self-produced matrix constituted by Extracellular Polymeric Substances (EPS) (Stark et al., 1999; Cole et al., 2004; Grande et al., 2020).

The biofilm represents a mechanism of microbial response to environmental stresses based on a cell–cell communication, interaction and cooperation to overcome the attack of antimicrobials and the host immune system (Hall-Stoodley et al., 2004; Flemming et al., 2016; Grande et al., 2020).

The EPS matrix of *H. pylori* biofilm is formed by proteomannans, LPS-related structures, extracellular DNA (eDNA), proteins, and Outer Membrane Vesicles (OMVs) (Grande et al., 2015; Hathroubi et al., 2018). The OMVs are spherical bilayered structures of 20–250 nm in diameter produced by Gram-negative bacteria and are involved in numerous mechanisms such as the promotion of bacterial survival, intercellular communication, delivery of virulence factors, biofilm development, horizontal gene transfer and modulation of the host immune response (Parker and Keenan, 2012; Orench-Rivera and Kuehn, 2016; Marinacci et al., 2023; Puca et al., 2024).

The important role of OMVs in *H. pylori* biofilm development and stability has been demonstrated (Yonezawa et al., 2009; Grande et al., 2015). Yonezawa et al. proved that the OMVs are a component of *H. pylori* biofilm extracellular matrix being involved in a cell–cell binding, defining the OMVs as “a potentially novel gastric cell colonization factor of *H. pylori*” (Yonezawa et al., 2009). In support of this thesis, recent articles demonstrated how OMVs released by *H. pylori* could induce gastric damage. Choi and co-authors hypotesized that *H. pylori*-derived OMVs could promote the development of various gastric diseases stimulating IL-8 production and NF-κB activation (Choi et al., 2021), while Melo and co-authors demonstrated that *H. pylori* OMVs induce modifications in gene expression and transcriptomic profile of gastric adenocarcinoma MKN74 cell lines. These changes were almost similar to those induced by the parental microorganism, amplifying the effects of the microorganism itself and suggesting that OMVs could probably induce them at sites distant from the primary infected organ (Melo et al., 2024).

Subsequently we detected eDNA associated with OMVs of *H. pylori*, and assumed that bOMVs had a structural role by preventing eDNA degradation, providing a bridging function between the vesicle surfaces and eDNA strands and promoting aggregation (Grande et al., 2015; Puca et al., 2019). The OMVs have been defined as an alternative mechanism of bacterial secretion; in fact, through the fusion with the membranes of both other bacteria and/or eukaryotic cells they guarantee the release of toxins into the host cell cytoplasm preventing their diffusion in the extracellular environment (Kadurugamuwa and Beveridge, 1999; Elluri et al., 2014; Jäger et al., 2015). The release of toxins into the host cells implicates cell functional impairment, DNA damage and in some cases cell death (Chitcholtan et al., 2008; Parker and Keenan, 2012; Chew et al., 2021).

*Helicobacter pylori* OMVs have been biochemically and functionally characterized. *H. pylori* OMVs contain *α*-carbonic anhydrase (Ronci et al., 2019) and the selective inhibition of *H. pylori* carbonic anhydrases by carvacrol and thymol compromises both biofilm production and OMVs release (Grande et al., 2021).

Moreover, Olofsson et al. demonstrated the presence of virulence factors involved in disease development as well as the adhesin proteins BabA and SabA or the oncoprotein CagA associated with the surface of OMVs (Olofsson et al., 2010). Recently, Peng and colleagues showed a pivotal role for OMVs in bacterial adhesion to host cells, detecting a significant enrichment of the N-terminal adhesion domain of SabA (Peng et al., 2024). Parker and colleagues demonstrated that the presence of VacA in *H. pylori* vesicles enhances cells internalization, and showed that VacA-OMVs are internalized via clathrin-mediated endocytosis, while VacA^+^ OMVs may use more than one pathway of internalization (Parker et al., 2010). Subsequently, Snider and colleagues studied the exoproteome of *H. pylori* at different time points and detected 74 proteins that are selectively released in the extracellular environment via OMVs, therefore, the composition of exoproteome was dependent on the bacterial growth phase (Snider et al., 2016).

Although characteristics of OMVs released in *H. pylori* planktonic phenotype (pOMVs) has been defined, no studies regarding the exoproteome of the biofilm phenotype (bOMVs) of this bacterium have been carried out. Therefore, the aim of the present study was a comparative analysis of *H. pylori* OMVs secreted in planktonic and biofilm form in order to evaluate possible differences in both eDNA presence and the exoproteome composition.

## 2 Materials and methods

### 2.1 Bacterial strains and media

The bacterial strain used in this study was the reference strain *H. pylori* ATCC 43629. The strain, stored at −80 °C before being thawed at room temperature, was plated on Chocolate Agar (CA) (Oxoid Limited, Hampshire, UK), supplemented with 1% (v/v) of IsoVitaleX (Becton Dickinson, Franklin Lakes, New Jersey, USA) and 10% (v/v) of defibrinated horse sterile blood (Oxoid Ltd), and finally incubated at 37 °C for 3 days in a microaerophilic atmosphere (Campy Pak Jar) (Oxoid Ltd).

### 2.2 Biofilm formation assay, cell viability evaluation and Confocal Laser Scanning Microscopy (CLSM) analysis

Bacteria were grown and biofilm was developed for 2, 6 and 10 days as previously described (Ronci et al., 2019). The broth cultures inoculated in 3.5 cm in diameter Petri dishes were used to test biofilm formation and cell viability by Live/Dead staining and Confocal Laser Scanning Microscopy (CLSM) analysis; while the broth cultures inoculated in 90 mm diameter petri dishes were used for OMVs detection and enumeration as well as analysis of planktonic and biofilm phenotypes. Broth cultures were also plated on CA in aerobic conditions to assess the absence of any contaminating microorganisms.

Biofilms cells viability was evaluated by CLSM using *BacLight* bacterial viability Kit (Life Technologies, Carlsbad, CA USA) according to the manufacturer’s instructions. CLSM acquisitions of SYTO 9 + Propidium Iodide (PI) stained biofilms, were performed using a Zeiss LSM 510 META confocal laser scanning system connected to an inverted Zeiss Axiovert 200 microscope and equipped with a Plan Neofluaroil oil-immersion objective (63X/1.4 NA; Zeiss international, Oberkochen, Germany). To separate the emission of the two dyes were used the main beam splitter HFT 488/543/633 and the primary NFT 635VIS and secondary NFT 545 dichroic mirrors. All the images were acquired by a sequential scan, using the 505–530 and 585–615 band-pass emission filters for the green and the red channels, respectively, to avoid spectral overlap. All experiments were performed at room temperature and each petri dish was used for no longer than 1 h. Results are the average of five independent experiments performed in duplicate.

### 2.3 Detection of bacterial morphology via fluorescence microscopy analysis

Cell shape distribution of *H. pylori* ATCC 43629 cells derived from 2, 6, and 10 day biofilm cultures was quantified post-microscopically using the ImageJ software version 1.54j. Following the biofilm formation described in the previous section, the cells were stained with SYTO 9 for 15 min and subsequently subjected to fluorescence microscopy analysis via Fluorescence Leica 4,000 DM Microscope (Leica Microsystems, Wetzlar, Germany). For this purpose, bacterial cells were classified as spiral/rod-shaped when their circularity was < 0.8, whereas cells with a circularity ≥ 0.8 were designated as coccoid forms, according to the criteria previously established by our group (Krzyżek et al., 2025). All cells from the observation fields were counted. For each time point, three biological repetitions in duplicate were made.

### 2.4 OMVs isolation

The pOMVs and bOMVs isolation from *H. pylori* was performed at each time point as reported elsewhere (Maccelli et al., 2020; Yu et al., 2024).

After incubation in Petri dishes (90 mm), planktonic cells were harvested, while the biofilm attached to the bottom was rinsed with PBS, and then scraped to collect the cells. Both planktonic and biofilm suspensions were centrifuged at 4 °C, 4000 rpm, for 20 min. Both supernatants were collected and sterile filtered with 0.2 μm filters to obtain cell-free samples that were further purified using a Beckman coulter Optima XL – 100 K ultracentrifuge (Beckman coulter, United States) at 50000 rpm, for 2 h at 4°C. Using this approach, authors are quite confident to remove cell debris or aggregates obtaining a good-quality crude OMVs extract. The obtained vesicle pellets were washed with PBS and ultracentrifuged again using the same parameters.

The pellets were dissolved in PBS and processed for Transmission Electron Microscopy (TEM), Nanoparticle Tracking Analysis (NTA), Dynamic Light Scattering (DLS) and Electrophoretic Light Scattering (ELS). The exoproteome was studied via nanoLC–MS/MS.

Moreover, the extracellular DNA (eDNA) associated with *H. pylori* OMVs was detected and quantified by using Quant-iT™ PicoGreen dsDNA assay kit (Thermo Fisher Scientific, Waltham, MA, USA) according to manufacturer’s instructions and as previously reported (Grande et al., 2017).

Colony Forming Units (CFU) count was carried out at each time points for both planktonic and biofilm phenotypes to determine the number of viable bacterial cells.

### 2.5 Transmission Electron Microscopy (TEM)

TEM analysis was carried out as previously described (Marinacci et al., 2025). Briefly, pOMVs and bOMVs in PBS suspension were distributed on a formvar–carbon–coated grid (Electron Microscopy Sciences, Hatfield, United Kingdom), and negatively stained with UAR solution. Samples were then analyzed with a JEM 1400 Flash TEM at 100 kV (Jeol, Tokyo, Japan) equipped with a sCMOS “Matataki” camera and SightX Viewer Software Ver.2.1.26.1818.

### 2.6 Nanoparticle Tracking Analysis (NTA), Dynamic Light Scattering (DLS) and Electrophoretic Light Scattering (ELS)

The OMVs were quantified by using NTA and physico-chemically analyzed via DLS and ELS, as previously described (Grande et al., 2017; Puca et al., 2019). The concentration of OMVs was directly tracked with the NanoSight PRO (NanoSight™ technology, Malvern-Panalytical, UK) while the DLS and ELS parameters were obtained with the Zetasizer PRO (Malvern-Panalytical, UK); the analyses were carried out at the ALFATEST laboratory (Milan, Italy), according to the company’s standard operating procedure: laser – 488 nm, capture duration [frames] – 750, number of captures – 5, flow rate [μL/min] – 3.0 for NTA; laser – 633 nm; number of measures – 3; backscattering angle – 173° for DLS; number of measures – 3; forward scattering angle – 13° for ELS. The number of vesicles was normalized to the number of bacterial cells, via the determination of OMVs/bacterial cells ratios.

### 2.7 Protein extraction and filter-aided sample preparation

The OMVs derived from three independent experiments were lysed by adding urea buffer 8 M, tris 10 mM, dithiothreitol 50 mM, SDS 2% and Triton X-100 0.4% and centrifuged at 13000 × *g* for 15 min to remove the insoluble fraction. The supernatant was transferred to a new tube, and the proteins were quantified through the Bradford assay. A volume corresponding to 30 μg of proteins was loaded onto a Nanosep 10-kDa-cutoff filter (Pall Corporation – Michigan, USA) and digested according to the protocol we routinely use in our laboratory, adapted from Distler (Distler et al., 2016; Di Giacomo et al., 2020).

### 2.8 LC–MS/MS label free shotgun proteomics

Each digested protein sample was analyzed in technical duplicate by LC–MS/MS using a Proxeon EASY-nLCII (Thermo Fisher Scientific, Milan, Italy) chromatographic system coupled to a Maxis HD UHR-TOF (Bruker Daltonics GmbH, Bremen, Germany) mass spectrometer (Di Giacomo et al., 2020).

### 2.9 Bioinformatics processing

Raw data were processed using PEAKS Studio v7.5 software (Bioinformatic Solutions Inc., Waterloo, Canada) using the ‘correct precursor only’ option. The mass lists were searched against a custom database containing reviewed and unreviewed *Helicobacter pylori* proteins downloaded from the Uniprot website^1^ to which a list of common contaminants was appended (1,810 entries). Carbamidomethylation of cysteines was selected as a fixed modification, and oxidation of methionines and deamidation of asparagine and glutamine were set as variable modifications. A maximum of 2 missed cleavages was allowed to one end of the peptides. Ten ppm and 0.05 Da were set as the highest error mass tolerances for precursors and fragments, respectively. Label free quantification analysis of identified proteins was carried out using the integrated tool PEAKS-Q, part of the PEAKS Studio suite.

Gene Ontology and Enrichment analysis were performed with the online tool ShinyGO v0.76^2^. ShinyGO is based on gene ontology (GO) annotation and gene ID mapping of 315 animal and plant genomes in Ensembl BioMart release based on Ensembl Release 104 with revision, archived on April 4, 2022. In addition, 115 archaeal, 1,678 bacterial, and 238 eukaryotic genomes are annotated based on STRING-db v10. *Helicobacter pylori* 26,695 STRINGdb was selected species.

Protein–protein interaction analysis and pathway enrichment analysis were performed with STRING v11.5^3^ using the standard settings.

### 2.10 Statistical analysis

Results represent the mean of three experiments ± S.D. (standard deviation). The statistical analysis of data was performed using ANOVA. To evaluate the statistical significance of data during the experimental results, *p*-value *<* 0.05 was used as the significance criterion.

## 3 Results

### 3.1 Biofilm characterization

**Figure 1:**
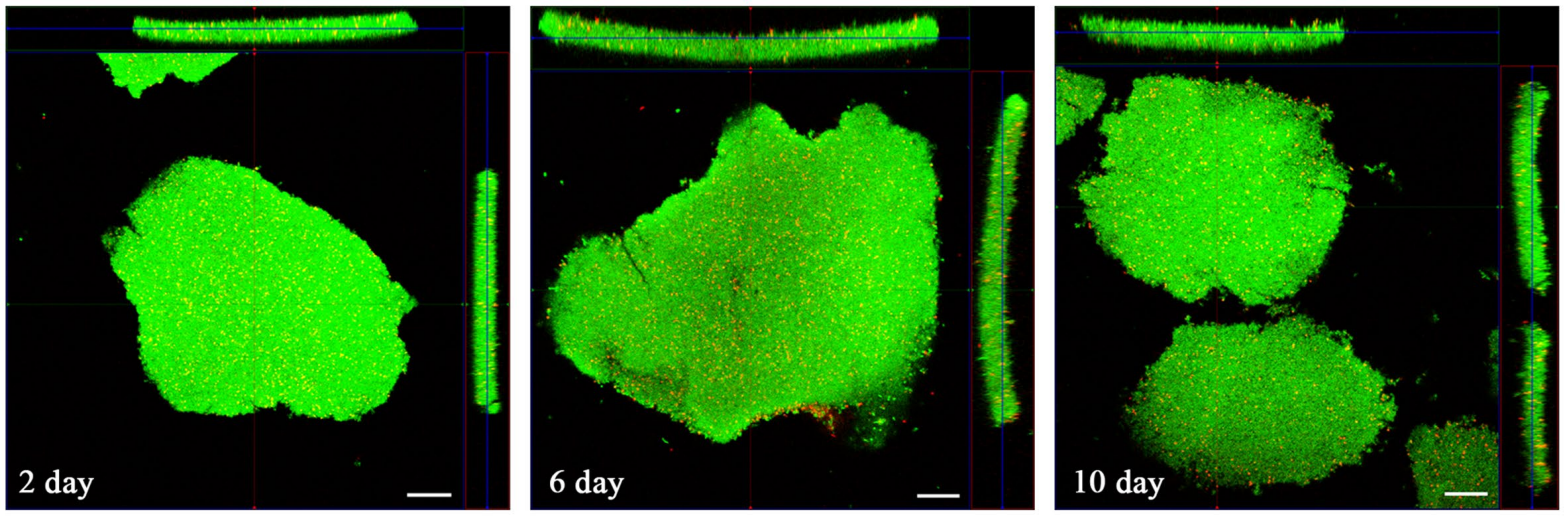
*Helicobacter pylori* biofilm formation over time. CLSM representative images of live/dead *H. pylori* (ATCC #43629) biofilm production at 2, 6, and 10 days of incubation. The images represent an orthogonal reconstruction (X-Z; Y-Z) of the biofilm stained with SYTO 9 (viable cells, green fluorescence) and PI (dead cells, red fluorescence). Scale bar = 10 μm. The image represents the average of five different experiments performed in duplicate.

**Figure 2:**
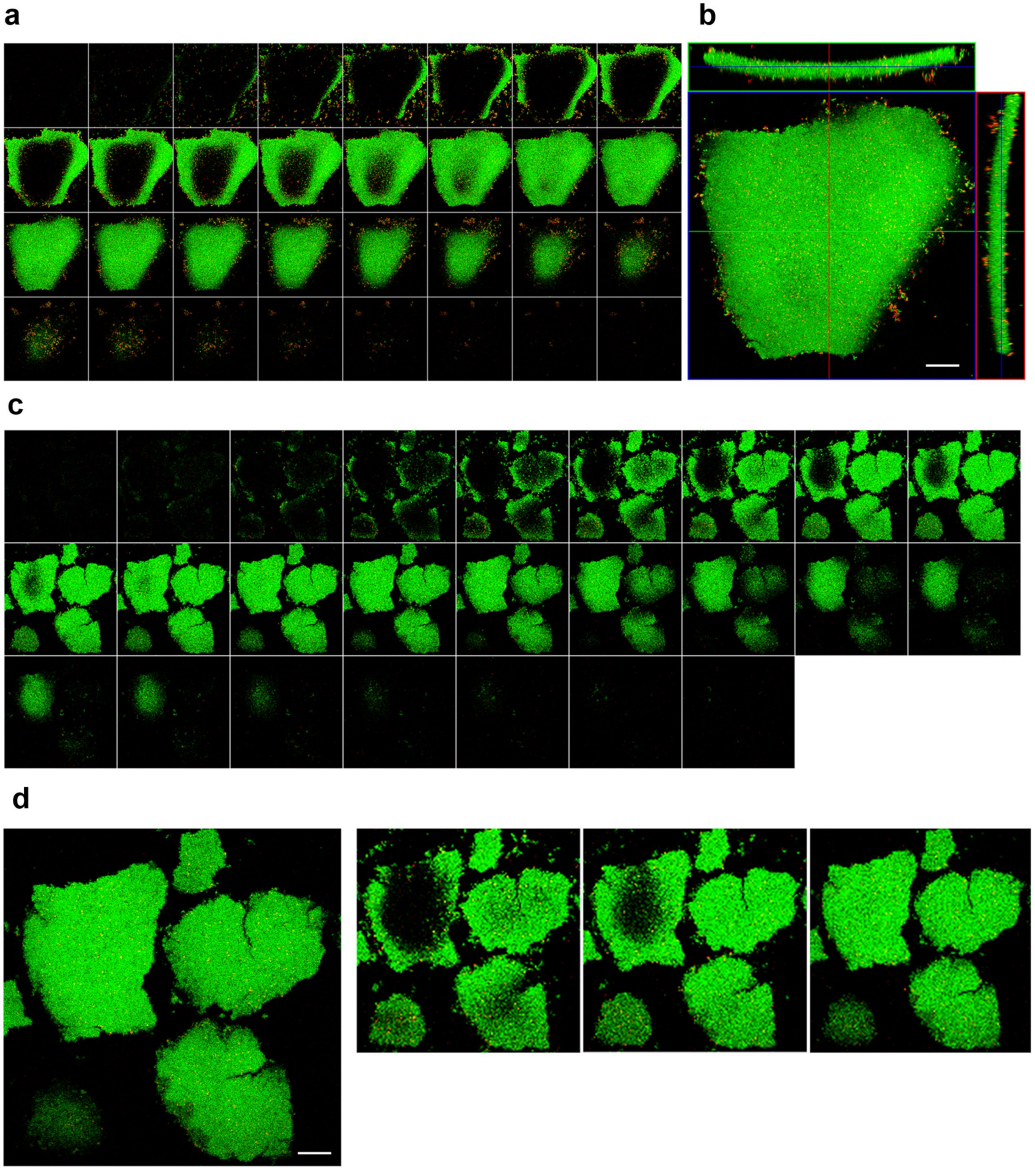
*Helicobacter pylori* biofilm. CLSM representative images of live/dead stained, 10 days old *H. pylori* (ATCC #43629) biofilm, where SYTO 9 showcases viable cells – green fluorescence and PI dead cells – red fluorescence **(a,c)**, Serial section gallery; **(b)** Orthogonal (X–Z; Y–Z) of the stained biofilm; **(d)** Representative image of a Z-stack from the bottom to the top of the biofilm. The images showed the presence of empty areas within clusters and water channels (scale bar = 20 μm).

Biofilm formation over time was confirmed by Live/Dead staining and CLSM analysis*. H. pylori* ATCC 43629 biofilm was characterized by 3D tower-like structures with a thickness of 20–30 μm heterogeneously interspersed with voids (Figure 1). The biofilm consisted mostly of live cells (green) and the number of dead cells (red) was negligible (Figures 1, 2a–d).

In 6 and 10 day-old *H. pylori* biofilms some fractures corresponding to water channels (Figures 2d) were detectable. Moreover, dark, empty areas were often visible within the larger clusters (Figures 2a,c,d).

Based on microscopic morphology assessment, it was determined that with increasing culture incubation time, *H. pylori* ATCC 43629 biofilm cells transformed from spiral to coccoid forms (Supplementary Figure 1). Specifically, the 2-day biofilm was composed primarily of spiral cells (87.5 ± 2.5%), whereas the 6- and 10-day biofilms were dominated by coccoid forms (98.6 ± 1.4% and 100 ± 0%, respectively).

### 3.2 TEM analysis

**Figure 3:**
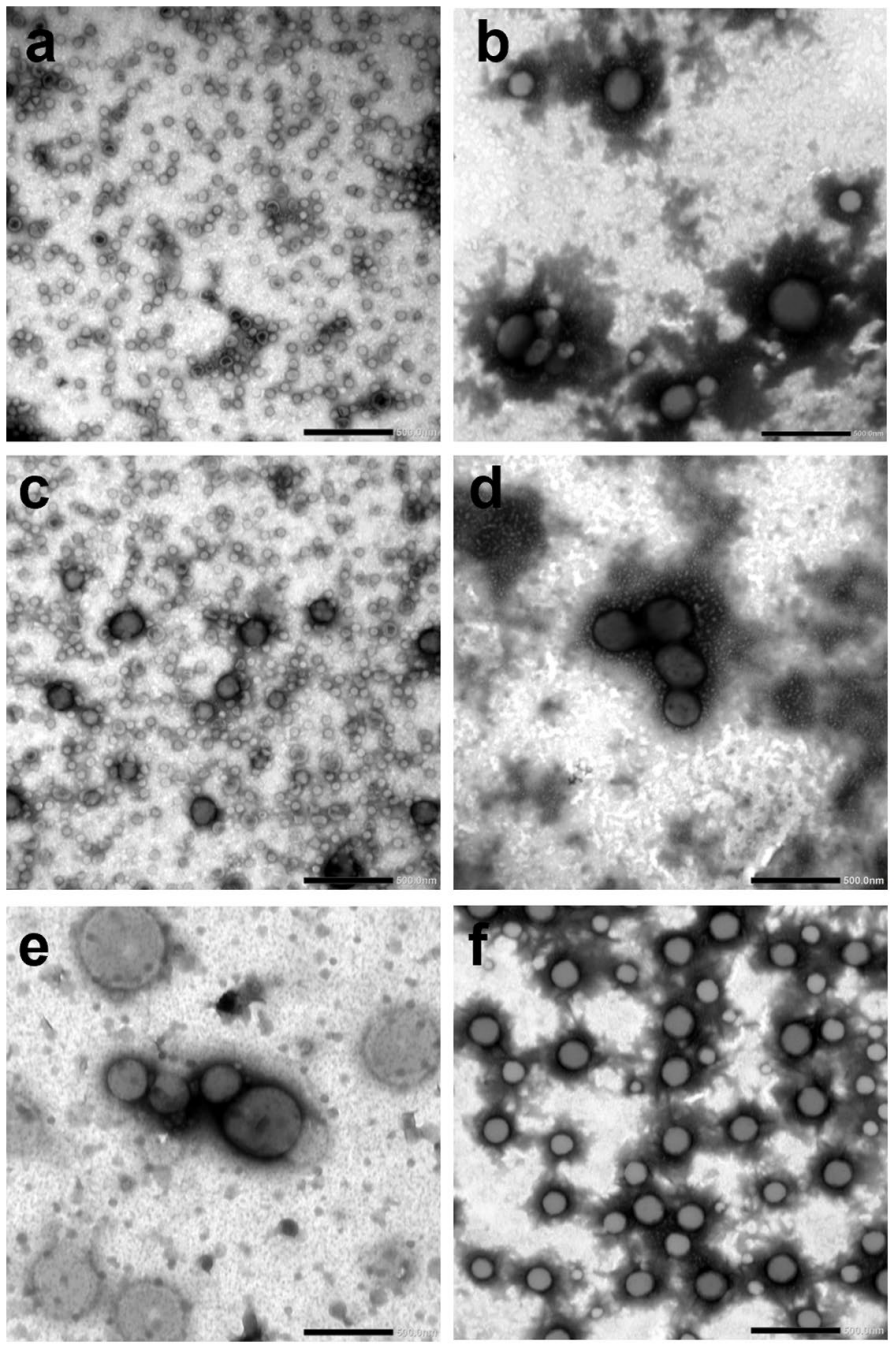
TEM analysis of negatively stained *H. pylori* OMVs at different times of incubation. **(a)** pOMVs released after 2 days of incubation; **(b)** bOMVs released after 2 days of incubation; **(c)** pOMVs released after 6 days of incubation; **(d)** bOMVs released after 6 days of incubation; **(e)** pOMVs released after 10 days of incubation; **(f)** bOMVs released after 10 days of incubation. Scale bar = 500 nm, magnification 15,000×.

*Helicobacter pylori* OMVs were analyzed via TEM to evaluate their morphology, size and integrity (Figure 3). *H. pylori* OMVs showed round shapes and intact surfaces. The vesicles from the planktonic phenotype are larger in size with prolonging incubation time. Differently, the vesicles from the biofilm phenotype produced over time showed more reproducible sizes. In detail, pOMVs at 2 and 6 days of incubation appear to be smaller than the biofilm counterpart (Figures 3a,c vs. Figures 3b,c); contrarily, pOMVs at 10 days of incubation (Figure 3e) are greater in size than all bOMVs (Figures 3b,d–f).

### 3.3 NTA, DLS, ELS analysis and PicoGreen assay

NTA allows to detect the size and the concentration of the nanoparticles in liquid aqueous suspension as reported in the table below which describes the concentration values and dimensional parameters, expressed in number base.

NTA analysis showed that the planktonic phenotype produced a greater number of vesicles compared with the biofilm phenotype (Table 1). The number of pOMVs increased from 2 to 6 days and decreased again at 10 days of incubation. On the contrary, the concentration of bOMVs remained constant up to 6 days with a slight increase at 10 days of incubation time.

**Table 1:** Concentration, mean diameter and mode (±SD) values obtained from NTA analysis.

**Sample**	**Vesicles/mL**	**Mean Diameter (nm)**	**Mode (nm)**
2 days pOMVs	1.31 × 10^15^ ± 9.94 × 10^13^	82.0 ± 4.34	57.5 ± 5.70
2 days bOMVs	9 × 10^10^ ± 1.5 × 10^10^	100.0 ± 4.38	67.5 ± 7.58
6 days pOMVs	1.67 × 10^16^ ± 9.23 × 10^15^	92.0 ± 6.06	62.5 ± 2.24
6 days bOMVs	4.05 × 10^10^ ± 2.16 × 10^10^	93.0 ± 4.55	72.5 ± 8.22
10 days pOMVs	6.41 × 10^14^ ± 6.83 × 10^13^	80.0 ± 2.28	67.5 ± 5.7
10 days bOMVs	8.9 × 10^11^ ± 6.74 × 10^11^	85.0 ± 5.22	52.5 ± 7.07

Data represents the mean of five measurements performed on the same preparation for each sample.

Furthermore, the mean diameter of OMVs was detected. It should be specified that the vesicles larger than 200 nm could not be detected given that in the procedure of OMVs isolation, *H. pylori* supernatant was filtered with 0.2 μm filters to remove all the bacterial cells and any potential cellular debris. NTA demonstrated that the pOMVs and bOMVs obtained after 2 days of incubation had a mean diameter of 82.0 and 100.0 nm, respectively; whereas 92.0 and 93.0 nm were the mean diameter of pOMVs and bOMVs isolated after 6 days of incubation, finally, the mean diameter of pOMVs and bOMVs after 10 days of incubation was 80.0 and 85 nm, respectively, (Table 1). These data confirm the investigation carried out via TEM (Figure 3).

**Figure 4:**
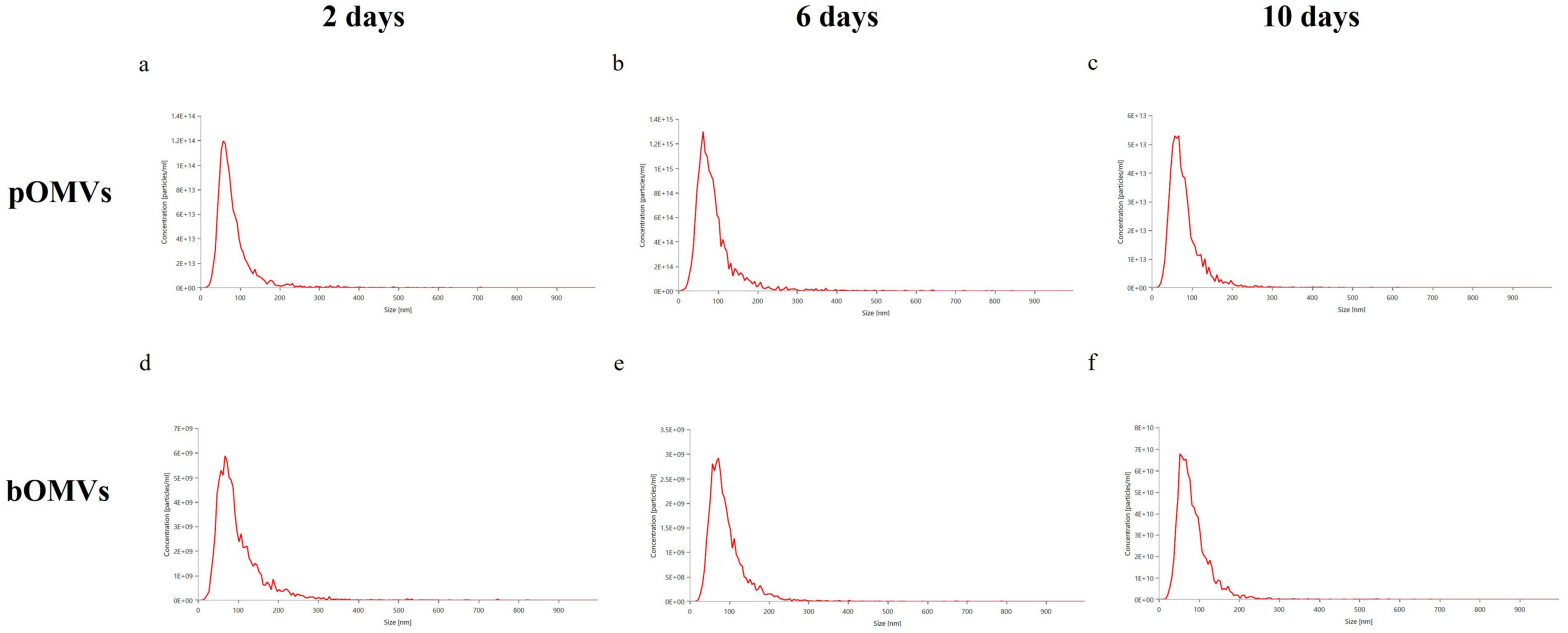
Average size distribution chart of *H. pylori* OMVs. **(a)** pOMVs released after 2 days of incubation; **(b)** pOMVs released after 6 days of incubation; **(c)** pOMVs released after 10 days of incubation; **(d)** bOMVs released after 2 days of incubation; **(e)** bOMVs released after 6 days of incubation; **(f)** bOMVs released after 10 days of incubation.

NTA analysis further showed that pOMVs at 2 and 10 days had the narrowest size distribution (Figures 4a,c); while pOMVs at 6 days and bOMVs obtained from the biofilm counterpart were more broadly distributed (Figures 4b,d–f) suggesting the presence of larger particles and different sub-populations. The changes over time in vesicle number, mean and modal diameters are statistically significant in both planktonic and biofilm phenotypes. Supporting this observation, all *p*-values were <0.05 with the sole exception for the modal size in planktonic phenotype (Supplementary Table 1).

Moreover, the total number of vesicles detected by NTA were normalized to the total number of bacterial cells determined by the CFU count and the results confirmed that planktonic cells produced more vesicles than biofilm ones at each time points (Supplementary Table 2).

**Figure 5:**
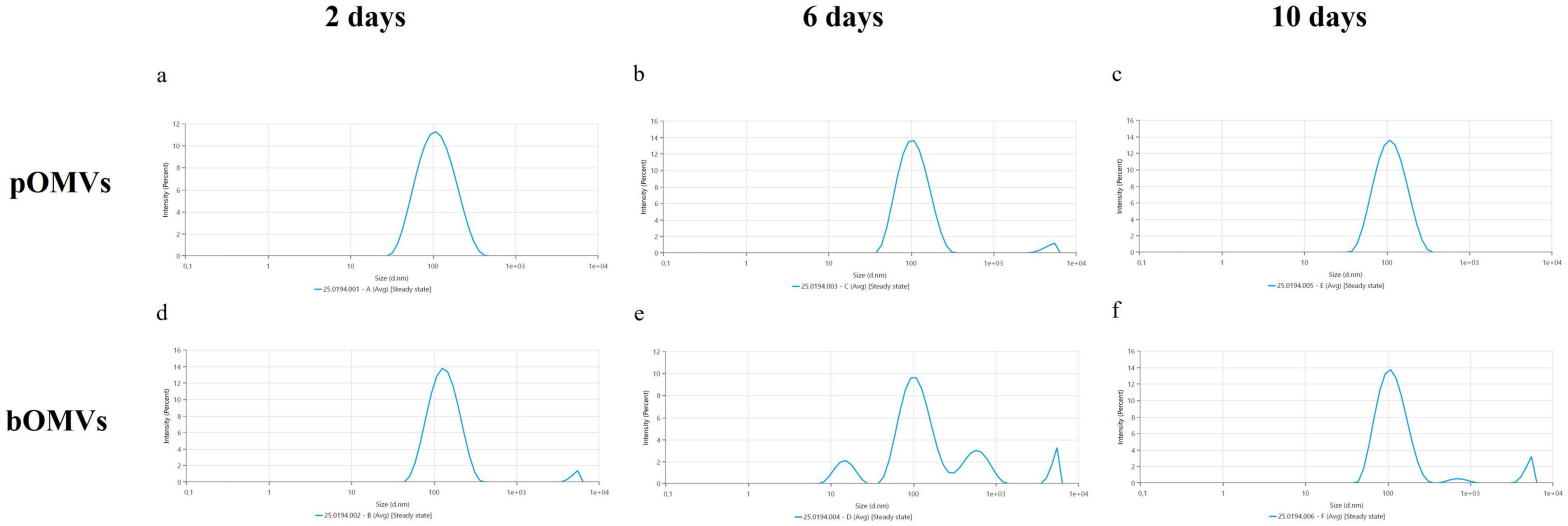
Size distribution by intensity. **(a)** pOMVs released after 2 days of incubation; **(b)** pOMVs released after 6 days of incubation; **(c)** pOMVs released after 10 days of incubation; **(d)** bOMVs released after 2 days of incubation; **(e)** bOMVs released after 6 days of incubation; **(f)** bOMVs released after 10 days of incubation.

Dynamic Light Scattering analysis partially confirmed the results obtained via NTA (Figure 5). In detail, DLS demonstrated that all the vesicles, except for bOMVs at 6 and 10 days, had a narrow size distribution and only one peak, corresponding approximately to 100 nm (Figure 5). bOMVs at 6 days resulted the most heterogeneous: two peaks (15 and 116,1 nm) suggested the existence of two different populations of vesicles, while the third peak (600 nm) likely indicated the presence of aggregates (Figure 5e). Similarly, bOMVs at 10 days presented a second peak that could be attributed to aggregation. As shown in Figure 5, four samples displayed peaks in the micrometer range. Authors speculate that they correspond to aggregates, given that the samples were filtered with 0.2 μm filters, as confirmed elsewhere (Barbieri et al., 2026). Additionally, the increased aggregation observed in OMVs isolated from the biofilm phenotype could be attributed to the intrinsic characteristics of the biofilm matrix, which comprises not only proteins and lipids, but also eDNA and polysaccharides (Grande et al., 2015; Hathroubi et al., 2018). In particular, polysaccharides could promote vesicle clustering via a mechanism known as polymer (or polysaccharide) bridging, whereby individual chains form physical links between vesicles. Moreover, the increase in local viscosity of the biofilm matrix promotes the likelihood of stable collisions between vesicles, further facilitating aggregation. Additionally, it is well known that *H. pylori* biofilm matrix contains eDNA that has a structural role providing a bridging function between OMVs surfaces. eDNA provides a bridging function between the OMVs surfaces, thus promoting vesicles aggregation (Grande et al., 2015; Hathroubi et al., 2018).

Overall, DLS data corroborated the PDI values, that were different among samples with a value over 0.4 only for bOMVs at 6 and 10 days of incubation (Table 2).

**Table 2:** *Z*-average and polydispersity index (PDI).

**Sample**	***Z*-average (nm)**	**PDI**	Peak 1*		Peak 2*		Peak 3 *	
			nm	%	nm	%	nm	%
2 days pOMVs	97.8	0.184	121.6	100	–	–	–	–
2 days bOMVs	119.6	0.266	139.4	97.5	5,047	2.5	–	–
6 days pOMVs	104.0	0.216	113.6	97.0	4,719	3.0	–	–
6 days bOMVs	123.0	0.473	116.1	67.8	600.1	17.6	15.0	9.4
10 days pOMVs	100.8	0.192	118.9	100	–	–	–	–
10 days bOMVs	116.4	0.412	117.6	92.1	5,027	5.8	723.7	2.1

The Z-potential values and electrophoretic mobility showed net negative charges for both bOMVs and pOMVs at different times of incubation (Table 3).

**Table 3:** Average results of Z potential and electrophoretic mobility.

Sample	Z-potential (mV)	Electrophoretic mobility (μmcm/*Vs*)
2 days pOMVs	–2.94	–0.23
2 days bOMVs	–3.15	–0.25
6 days pOMVs	–2.18	–0.17
6 days bOMVs	–3.26	–0.26
10 days pOMVs	–2.95	–0.23
10 days bOMVs	–2.57	–0.20

Furthermore, the eDNA associated to *H. pylori* OMVs over time was quantified via PicoGreen assay. PicoGreen labeled not only the eDNA but also the total dsDNA present in intact OMVs from planktonic and biofilm phenotypes. The results demonstrated that eDNA presence in pOMVs remained constant over time, in the range of 0.3–0.57 ng/μL, whereas the eDNA concentration was higher in bOMVs at 2 days of incubation in respect to 6 and 10 days, in which a lower concentration was detected (Supplementary Figure 2).

### 3.4 Shotgun proteomics identifies protein variation over time

**Figure 6:**
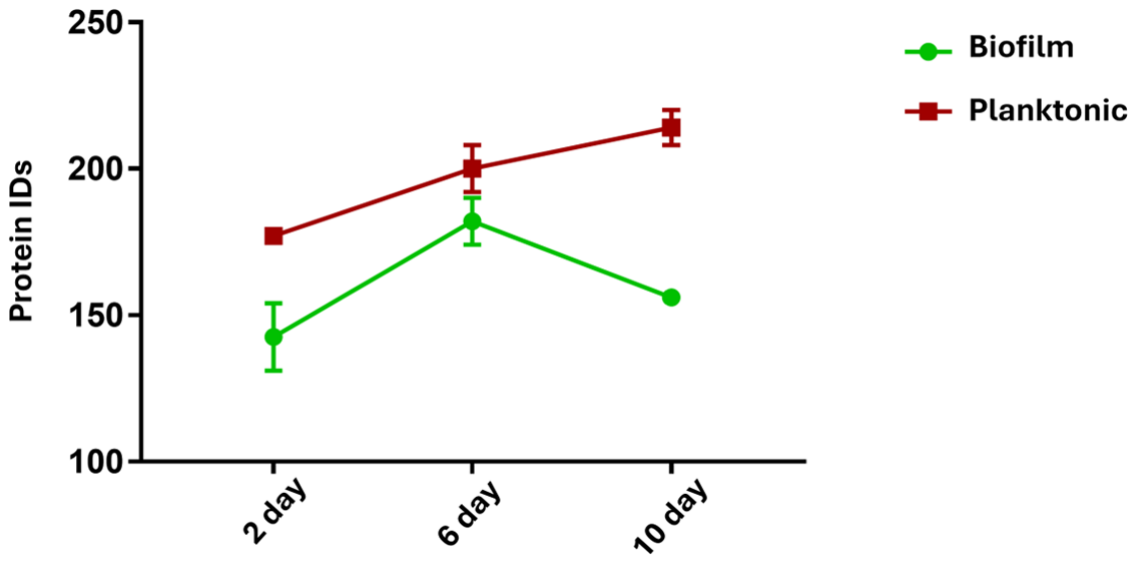
Number of total identified proteins for the two phenotypes of *H. pylori*. The graph shows the total number of identified proteins (IDs) in biofilm and planktonic phenotypes at each time point (2, 6, and 10 days). The number of protein IDs increases over time, and it is always higher for the planktonic phenotype.

In this study, a label-free shotgun proteomics approach was used to detect the variation over time of the cargo protein content of OMVs produced by two different phenotypes of *H. pylori*. Two technical replicates for each biological replicate at every time point were used. A moderate increase in the number of proteins identified was observed with time (2, 6, and 10 days) for both phenotypes. A number of proteins ranging from 131 to 220 for a single replicate were identified, setting the false discovery rate (FDR) at the peptide-spectrum matches (PSM) level to 0.1%, resulting in FDR at the protein level lower than 2%. As shown in Figure 6, the number of identified proteins was higher for the Planktonic phenotype at every timepoint.

A relative quantification analysis of the identified proteins for biofilm and planktonic phenotypes over a 2 to 10 days period, was performed through the label-free quantification module PEAKS-Q, part of PEAKS Studio 7.5. This represents a quantitative method based on the relative areas of the extracted ion chromatograms of peptides detected in multiple samples and applies the expectation–maximization algorithm to detect and resolve overlapping features. The features of the same peptide from different samples are aligned using a high-performance retention time alignment algorithm. The resulting differentially expressed proteins were filtered by considering only those identifications exhibiting a good replication rate (that is, identified in at least 33% of the replicates) and with significance ≥ 20 for proteins and fold change for proteins ≥ 1.5. Protein contaminants were manually removed from the ID lists. After filtering, 45 and 123 differentially expressed proteins were identified in biofilm and planktonic phenotypes, respectively (Table 4).

**Table 4:** List of proteins modulated between the Biofilm and Planktonic phenotypes identified by proteomic analysis.

**Biofilm phenotype**		
**Gene name**	**6days_vs_2days_Ratio**	**10days_vs_2days_Ratio**
ahpC	1.71	2.56
dps	3.57	5.58
ftnA	7.07	5.87
glnA	7.94	7.70
gltA	7.32	7.96
groL	3.18	6.15
groS	4.57	4.32
HP_0097	3.48	2.76
HP_0231	4.56	3.17
HP_0305	8.21	6.79
HP_0470	5.83	2.51
HP_0486	13.82	11.57
HP_0558	12.53	10.21
HP_0596	6.43	4.85
HP_0599	6.06	3.37
HP_0605	47.12	23.63
HP_0695	3.23	4.38
HP_0706	4.45	3.55
HP_0896	5.78	4.13
HP_0913	3.17	3.47
HP_1110	50.00	50.00
HP_1118	5.81	5.03
HP_1125	50.00	50.00
HP_1177	7.09	6.10
HP_1243	5.94	4.62
HP_1286	5.50	3.62
HP_1350	4.57	2.68
HP_1,400	6.01	3.41
HP_1454	4.88	4.71
HP_1501	7.65	2.54
HP_1512	8.67	8.99
HP_1562	12.79	8.48
HP_1588	11.52	8.97
hp1018/19	3.96	2.96
hpaA	50.00	50.00
katA	4.74	9.45
lpp20	6.57	7.41
ribH	14.53	17.70
rpoBC	0.68	0.22
sodB	50.00	50.00
trxA	5.04	5.48
ureA	3.91	7.33
ureB	3.36	6.72
ureB	4.30	6.05
vacA	14.33	5.55
		

**Plankton phenotype**		
**Gene name**	**6days_vs_2days_Ratio**	**10days_vs_2days_Ratio**
ahpC	0.99	0.20
amiE	50.00	50.00
aspA	50.00	50.00
atpC	1.01	0.01
C694_06140	0.57	0.23
cdh	0.46	0.01
dps	2.35	2.50
fba	50.00	50.00
flaA	0.59	0.01
frdA	0.52	0.01
ftnA	0.92	0.11
fusA	50.00	50.00
glnA	6.01	3.34
groL	5.21	12.84
groS	2.32	0.66
hcpA	1.89	0.01
hcpC	1.65	0.01
hcpD	5.06	1.68
HP_0018	4.75	3.12
HP_0087	1.38	0.25
HP_0097	1.17	0.69
HP_0122	50.00	50.00
HP_0127	4.89	2.42
HP_0129	1.64	1.29
HP_0130	1.79	0.35
HP_0135	11.88	7.65
HP_0175	2.13	1.49
HP_0204	3.01	0.89
HP_0227	3.39	2.50
HP_0229	1.96	0.37
HP_0231	1.63	1.22
HP_0232	4.28	0.12
HP_0260	2.47	0.14
HP_0275	0.78	0.26
HP_0305	1.86	1.17
HP_0317	1.69	0.70
HP_0377	3.04	1.93
HP_0397	50.00	50.00
HP_0408	2.52	1.24
HP_0410	2.09	0.93
HP_0472	3.60	0.25
HP_0485	1.04	0.15
HP_0486	3.90	2.32
HP_0589	4.20	11.25
HP_0590	3.21	9.26
HP_0596	3.80	3.94
HP_0605	1.04	0.35
HP_0645	1.17	1.88
HP_0657	1.04	0.01
HP_0659	1.42	0.93
HP_0671	4.35	1.78
HP_0686	4.66	1.65
HP_0694	3.88	1.88
HP_0695	4.02	7.73
HP_0696	4.22	13.82
HP_0697	7.07	10.46
HP_0710	2.36	0.02
HP_0719	1.70	0.66
HP_0721	2.11	1.87
HP_0746	2.34	0.96
HP_0758	1.22	0.01
HP_0783	2.58	1.69
HP_0788	1.81	0.54
HP_0836	50.00	50.00
HP_0837	5.09	3.20
HP_0896	2.57	3.01
HP_0912	2.94	2.82
HP_0913	1.62	1.35
HP_1012	1.27	0.23
HP_1037	50.00	50.00
HP_1104	15.98	46.61
HP_1,111	2.57	4.40
HP_1118	1.67	0.01
HP_1125	2.42	0.64
HP_1167	2.80	0.01
HP_1172	1.51	0.96
HP_1173	3.31	1.94
HP_1177	1.67	0.86
HP_1193	6.95	2.87
HP_1227	3.08	0.01
HP_1243	2.36	1.69
HP_1285	1.77	1.08
HP_1286	1.92	0.76
HP_1287	35.63	75.66
HP_1326	2.57	1.32
HP_1350	1.04	0.42
HP_1395	2.01	0.01
HP_1,400	1.56	0.60
HP_1453	2.49	0.16
HP_1454	6.69	0.98
HP_1455	50.00	50.00
HP_1457	3.37	1.18
HP_1461	2.91	1.49
HP_1,462	2.83	0.35
HP_1469	3.83	2.53
HP_1489	1.71	0.01
HP_1501	1.51	0.34
HP_1512	2.10	1.02
HP_1524	50.00	50.00
HP_1561	2.22	1.09
HP_1562	1.76	0.95
hp1018/19	2.65	2.33
hpaA	2.73	3.01
katA	1.88	0.78
lpp20	3.43	3.28
msrAB	0.93	0.20
pdxJ	3.31	0.71
pepA	2.86	0.06
pgbA	2.67	0.95
pgbB	2.10	0.11
ribH	2.79	0.12
rlpA	4.68	1.46
rnj	1.57	0.01
rplB	87.82	53.63
rpoBC	6.54	6.10
rpsC	2.16	1.64
rpsG	7.35	6.01
tolB	1.46	0.54
tuf	5.46	15.28
ureA	6.65	6.12
ureB	3.21	1.63
ureB	4.57	2.44
vacA	0.76	0.65

Modulation is expressed as a ratio between 6 and 10 days vs. 2 days (to avoid values of infinite ratio derived by proteins not identified at 2 days, the arbitrary value of 50.00 was chosen).

The proteins found in OMVs released from the biofilm phenotype were all almost detected in the planktonic phenotype. Interestingly, for the biofilm phenotype, all the proteins increased their expression after 6 days with some of them decreasing slightly at 10 days with a sort of peak effect. The only exception to this general trend was RpoBC protein (Bifunctional DNA-directed RNA polymerase subunit beta/beta), a DNA-dependent RNA polymerase that catalyzes the transcription of DNA into RNA using the four ribonucleoside triphosphates as substrates. The downregulation of this protein has been recently reported to be associated with metronidazole sensitivity. Conversely, for the planktonic phenotype, many proteins decreased after 6 days and even further at 10 days. It is worth noting the presence of many outer membrane proteins as well as uncharacterized proteins in both phenotypes. Interestingly, for the planktonic phenotype, VacA did not vary substantially at 6 days and 10 days compared to 2 days. Conversely, for the biofilm phenotype, VacA was highly overexpressed at 6 days and 10 days. LPP20, a membrane-associated lipoprotein, which is believed to play a role in the pathogenesis of *H. pylori* by serving as an inflammatory mediator, is overexpressed in both phenotypes at 6 and 10 days.

### 3.5 Enrichment analysis

**Figure 7:**
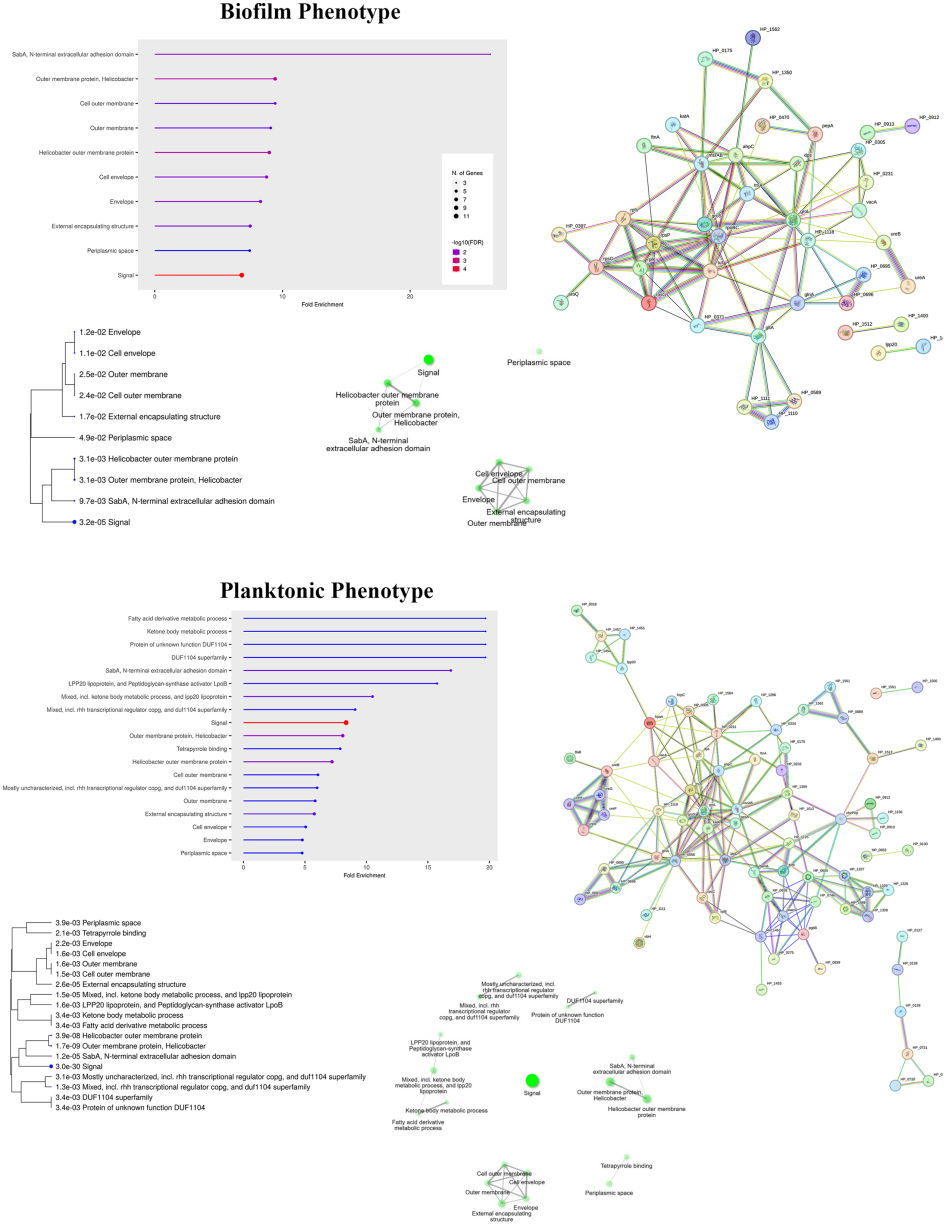
Enrichment analysis of the differentially expressed proteins for biofilm and planktonic phenotypes with relative tree view in which a hierarchical clustering of the pathways is shown. A network plot showing the relationship between enriched pathways is also shown for each phenotype. Two pathways (nodes) are connected if they share 20% or more genes. Darker nodes are more significantly enriched gene sets. Bigger nodes represent larger gene sets. Thicker edges represent more overlapped genes. Protein–protein interaction network is also shown on the right.

The dataset of proteins with the corresponding expression ratios “6 days vs. 2 days” and “10 days vs. 2 days” for the biofilm and planktonic phenotypes were submitted to enrichment analysis using the online tool ShinyGO version 0.76 (see footnote 2) selecting *Helicobacter pylori* 26,695 species and setting the FDR cutoff to 0.05%, and showing only 20 pathways with minimum pathway size of 2 (Figure 7) (Ge et al., 2020). The most significantly enriched pathways are related to SabA, N-terminal extracellular adhesion domain and outer membrane related proteins for the biofilm phenotype and to fatty acid derivative metabolic process and protein of unknown function (DUF1104), for the planktonic phenotype. In both phenotypes, the signal pathway resulted in the highest enrichment FDR. Interestingly, the protein of unknown function (DUF1104) is a protein family of several hypothetical proteins of unknown function reported to be largely found in *Helicobacter pylori*.

Protein–protein interaction analysis using STRING v11.0 (see footnote 3) was also performed using the standard settings. The resulting networks were imported in Cytoscape v. 3.9.1 for visualization and are shown in Figure 7 (right). Each node represents a protein with splice isoforms or post-translational modifications collapsed to a single protein-coding gene. Each edge represents a protein–protein association. Association terms derive from known and predicted interactions, as well as from text mining, co-expression, and protein homology, and they are meant to be specific and meaningful. Notably, the results of this analysis showed that the majority of the proteins (42 over 45 for the biofilm and 118 over 123 for the plankton submitted proteins) are connected within a single network, with a highly significant protein–protein interaction enrichment (*p*-value < 10^−16^).

## 4 Discussion

The microbial biofilm is a structure that determines a number of beneficial features for its producers, including protection against antimicrobial substances and the immune system (Hall and Mah, 2017; Jiang et al., 2020). For a long time since the discovery of *H. pylori*, it was thought that this bacterium was incapable of producing biofilm or able to do it only in *in vitro* conditions (Krzyżek et al., 2020). Currently, however, more attention in scientific research is paid to the biofilm phenotype of *H. pylori* and its importance in tolerance/resistance to antimicrobial drugs (Hathroubi et al., 2018; Krzyżek et al., 2020; Puca et al., 2023). In response to the need to deepen the knowledge on biofilm produced by *H. pylori*, this original paper provided information about the time-lapse formation of this biostructure. The 2-days biofilm of *H. pylori* was 20–30 μm thick and was characterized by tower-like structures. CLSM analysis showed a lack of significant changes in biofilm thickness over incubation time as previously demonstrated both in stationary and microfluidic conditions (Krzyżek et al., 2022). It seems that one of the important mechanisms responsible for this phenomenon is the ability of *H. pylori* to transform massively into viable but-nonculturable coccoid forms during a prolonged culture (Ierardi et al., 2020; Krzyżek and Grande, 2020). This morphotype is not able to multiply, although it is particularly strongly associated with the establishment of biofilms. Interestingly, in the late growth stages of *H. pylori* biofilm the presence of heterogeneously distributed water channels and voids was noted. The function of these structures in *H. pylori* physiology has not yet been described. Based on the reports of others (Quan et al., 2021), we suspect that water channels within biofilms may be involved in the removal of microbial secondary metabolites and facilitation of access to nutrients for deeper parts of highly developed microbial communities. Such functions have been shown for water channels produced by biofilm forms of, e.g., *Pseudomonas aeruginosa* (Davey et al., 2003), *Bacillus subtilis* (Wilking et al., 2013) or *Escherichia coli* (Rooney et al., 2020). Focusing on the presence of voids in mature *H. pylori* biofilm samples, it seems that they may be created as the result of partial dispersion of microbial biofilm and constitute a mechanism aimed at searching for new, nutrients-enriched niches (Rumbaugh and Sauer, 2020). The phenomenon of *H. pylori* dispersion is currently extremely poorly characterized and it is only known that the modulators of this process are autoinductors-2 (AI-2), being self-produced compounds involved in quorum-sensing of this bacterium (Anderson et al., 2015; Sweeney et al., 2019). The deepening of the subject regarding transition of *H. pylori* from the biofilm to the dispersion phase is undoubtedly an interesting scientific area that is worth further evaluation. Relationship between the voids production and microbial dispersion has already been widely investigated for some microorganisms such as *P. aeruginosa* (Huynh et al., 2012; Amari et al., 2013; Goodwine et al., 2019), but to our knowledge this is the first report describing *H. pylori* biofilm at a 10-day maturation stage and therefore the first to document the presence of such pronounced voids in advanced-stage biofilm of this bacterium.

Key structural components of *H. pylori* biofilm are OMVs, that can be released by this bacterium in both planktonic and biofilm phenotypes. Yonezawa et al. demonstrated that the TK1402 strain produces OMVs that directly promote biofilm formation and subsequent analyses of OMVs-associated proteins further confirmed their role in modulating biofilm architecture (Yonezawa et al., 2009, 2011). More recent studies showed that specific OMVs-associated proteins, such as AlpB, influence biofilm organization and cell adhesion in a strain-dependent manner (Yonezawa et al., 2017). Additionally, Krzyżek et al. (2022) showed that strong biofilm-producing strains have significantly higher amounts of OMVs than weak strains, as well as a more complex matrix and denser cell packing, indicating a marked structural remodeling associated with clarithromycin resistance. Overall, this evidence suggest that the presence of voids in 10-days biofilms may reflect an advanced stage of maturation, and it is plausible that OMVs may contribute to their formation or maintenance by modulating matrix organization and facilitating microbial dispersion.

It is now clear that such OMVs are relevant players in bacterial pathogenesis (Kaparakis et al., 2010; Turner et al., 2018; Marinacci et al., 2023; Puca et al., 2024) and carry proteins associated with translation and virulence factors (Mullaney et al., 2009). Therefore, OMVs may also promote bacterial survival and induce infection of the gastric epithelium (Chew et al., 2021). In this research article, the NTA was carried out to enumerate *H. pylori* OMVs as well as to determine their size. The number of the pOMVs increased from 2 to 6 days, similar to that described for OMVs isolated from *H. pylori* 26695 cultures at 24, 64 and 72 h (Zavan et al., 2019; Melo et al., 2021). Interestingly, both the bOMVs concentration and size remain constant over time, suggesting a sort of regulation in the OMVs release as well as in the biofilm stabilization. More in depth studies should be carried out to determine the modulation of the expression of genes associated with blebbing in both phenotypes.

The analysis of the proteome of pOMVs and bOMVs contributes to better understand the role of such structure, in the colonization, pathogenesis and virulence of the microorganism over time. The data obtained in this paper demonstrated that the proteome of pOMVs was more diverse and underwent greater dynamics over time of bacterial culture than that associated with biofilm (bOMVs). Among 123 pOMVs proteins classified as significantly changed in the expression, more than half of them (66 proteins) increased at both analyzed time points (6 days and 10 days vs. 2 days), while the other part decreased at both experimental stages (9 proteins) or had tendency to rise during the initial bacterial growth phases (2–6 days) with a subsequent decline during later culture stages (6–10 days) (48 proteins).

Among the pOMVs proteins of late stationary phases showing the most significant decrease in comparison to those isolated from the logarithmic bacterial growth, components related to virulence and resistance to antibiotics can be highlighted. In the first case, the most important representatives were toxins and lytic enzymes, i.e., VacA (Caso et al., 2021), HP_0657 (collagenase YmxG) (Marques et al., 2021), HP_1118 (*γ*-glutamyltranspeptidase) (Zhang et al., 2015), and PgbA (plasminogen-binding protein A) (Jönsson et al., 2004). This group was also represented by some adhesins early associated with gastric colonization, including HP_0710 (HomA), HP_1453 (HomD), HP_1167 (HofH) and HP1395 (HorL) (Xu et al., 2020). It appears that all the above-mentioned factors may be of crucial importance in the initial stages of infection, and are, therefore, highly expressed and packed into membrane vesicles of the logarithmic phase. The second group of proteins expressed at low levels in pOMVs isolated from the late stationary phase of bacterial cultures was associated with resistance to antimicrobial substances with representatives such as HcpA and HcpC (subunits of *β*-lactamase) (Tseng et al., 2009) and two potential representatives of efflux pumps (HP_0758 and HP_1489) (Raj et al., 2021). Similar results were obtained by Johnston and colleagues, who isolated pOMVs from a 16 h *H. pylori* 26695 broth culture grown in different acidic conditions. In detail, they observed that, during all growth conditions, HcpA, HcpC and HcpD proteins were more expressed in pOMVs compared to parental bacteria indicating that vesicles could play a role in enhancing bacterial survival during the early stages of colonization (Johnston et al., 2024). At first glance, it may seem surprising that bacteria isolated from the stationary phase, classically linked with higher antibiotic tolerance/resistance than the logarithmic phase (Brauner et al., 2016; Jaishankar and Srivastava, 2017), secrete OMVs containing fewer proteins with such a protective activity. However, together with the observation of other authors, we can hypothesize that these proteins may have a great protective role for spiral-shaped bacterial cells in the logarithmic phase, while they become less useful in the later stages of growth (6 and 10 days of incubation) when *H. pylori* tends to transform into coccoid form. This morphotype is characterized by changed physiology (Krzyżek and Gościniak, 2018; Krzyżek and Grande, 2020) which determines the tolerance to high concentrations of antibiotics (Faghri et al., 2014; Kadkhodaei et al., 2020). Hence, we believe that the secretion of these protective proteins in OMVs could be viewed as an unnecessary energy expenditure for coccoid forms of this pathogen. Nevertheless, the hypothesis concerning the changes in the resistome of OMVs and the association of this process with the morphological transition of *H. pylori* requires research evaluation and will be verified by us in the future.

Among the proteins with a high prevalence within pOMVs from the late phases of *H. pylori* growth, components related to nitrogen and carbon metabolism should be outlined. Factors involved in nitrogen metabolism were represented by UreA and UreB (Williams et al., 1996), AmiE (Skouloubris et al., 2001) and AspA (Leduc et al., 2010). Although these proteins are classically perceived to be involved in neutralizing the acidic pH of the stomach environment (Miller and Maier, 2014), their key role in the maintenance of nitrogen balance and amino acid synthesis by *H. pylori* is also indicated (Williams et al., 1996; Skouloubris et al., 2001; Leduc et al., 2010). This scenario seems to be confirmed by our observation showing the enrichment of proteins related to these processes within pOMVs with examples including an enzyme responsible for the serine synthesis - HP_0397 (D-3-phosphoglycerate dehydrogenase) (Grant, 2018), as well as components responsible for protein synthesis, such as elongation factors FusA and Tuf and one of the subunits of 50S ribosome – RplB (50S ribosomal protein L2) (Bhattacharyya et al., 2001). Carbon metabolism, on the other hand, was represented by Fba (fructose-bisphosphate aldolase) (Fonvielle et al., 2008), HP_1104 (cinnamyl-alcohol dehydrogenase (Cad)) (Mee et al., 2005), HP_1287 (aminopyrimidine aminohydrolase (TenA)) (Barison et al., 2009) and HP_0695-HP_0697 (an acetone metabolism operon encoding an acetone carboxylase subunits) (Brahmachary et al., 2008). According to the observations of other authors, OMVs can be considered as an effective long-range secretion system, one of the goals of which is to break down nutrients, process them and supply important metabolites to producers of these structures (Zakharzhevskaya et al., 2017; Salvachúa et al., 2020). The increase in expression of these proteins in pOMVs of late growth phases may most likely be related to the high density of bacteria and the need to activate alternative mechanisms of nutrients uptake. These observations are again consistent with results obtained by Zavan et al. (2019), who demonstrated that OMVs from late stages of bacterial growth were highly enriched in proteins involved in biosynthetic and metabolic pathways (Zavan et al., 2019). It seems that this mechanism may coexist with the previously discussed phenomenon of secretion of lytic enzymes and toxins in pOMVs from the logarithmic bacterial growth phase. In this scenario, early growth phase *H. pylori* cells secrete OMVs containing multiple virulence factors associated with host cell destruction and then during later growth stages these bacteria may produce another subpopulation of OMVs containing factors involved in the metabolic processing of these components. In our opinion, the numerous presence of proteins involved in the acetone metabolism deserves additional attention. Currently, little is known about the function of these proteins in the *H. pylori* physiology (Brahmachary et al., 2008), while the recently published research by Hathroubi et al. (2020), focused on the proteome of this pathogen and suggested that this operon is one of the most important in biofilm formation of *H. pylori*. In the present article, a time-dependent increase in the expression of these proteins in pOMVs (both with respect to 6 days vs. 2 days and 10 days vs. 2 days) was noticed. Based on this, it can be concluded that metabolic changes towards the acetone-dependent metabolism may be an important step in transition of *H. pylori* from planktonic to biofilm phase. It therefore seems that these proteins may constitute an attractive target for new therapies limiting the development of this biostructure. Undoubtedly, this is an element worth future research evaluation.

When discussing *H. pylori* biofilm, it is important to pay attention to the proteomic results of bOMVs obtained in the present article. In contrast to pOMVs, the proteome of bOMVs was much more homogeneous and stable over time with 44 out of the 45 proteins identified as being highly expressed. The reason could lie in the ability of *H. pylori* to activate molecular patterns that promote proteome stabilization and persistence in the biofilm phenotype. A major expression of stress-response proteins, chaperones, outer membrane components, and adhesion factors is related to the biofilm phenotype rather than planktonic one. In the biofilm phenotype, the pH sensitive *H. pylori* ArsRS two-component system (TCS) modulates acid adaptation and stress survival whereas the ferric uptake regulator Fur correlates iron availability with oxidative stress responses (Ernst et al., 2005; Servetas et al., 2018). Biofilm formation stabilizes secreted protein composition and improves membrane protein homeostasis via the stabilization and localization of adhesins (e.g., BabA, SabA) in the membrane. On the other hand, the planktonic phenotype, characterized by a greater proteome variability, correlates with faster protein turnover and fluctuating expression of motility proteins such as those related to flagella and higher transcriptional dynamism (Niehus et al., 2002; Pernitzsch and Sharma, 2012).

Among bOMVs-associated proteins, RpoBC (bifunctional DNA-directed RNA polymerase subunit beta-beta’) was the only one whose abundance varied with the culture time of *H. pylori* biofilm (Zakharova et al., 1998), exhibiting a gradual decrease. Among proteins that were abundant in bOMVs in late growth stages there were proteins mainly involved in iron accumulation, protection against oxidative stress, and virulence promoting tumorigenesis. The first group of proteins was represented by FtnA (bacterial non-heme ferritin), FecA (iron(III) dicitrate transport protein), Dsp (Hp_0243/NapA, neutrophil-activating protein A), HP_1512 (iron-regulated outer membrane protein (FrpB)), HP_1562 (iron(III) ABC transporter, periplasmic iron-binding protein (CeuE)) and RibH (6,7-dimethyl-8-ribityllumazine synthase), all of which aim is to facilitate transmembrane transport and/or accumulation of iron cations by *H. pylori* (Pich and Merrell, 2013; Gaddy and Haley, 2015). Literature data on various classes of microorganisms confirm our observations showing the ability of OMVs secreted by bacteria to bind iron ions (Lin et al., 2017; Li et al., 2021) and indicating the existence of a strong relationship between biofilm formation and availability of iron ions in the environment (Kang and Kirienko, 2018; Rizzi et al., 2018; Chen et al., 2020; Dauros-Singorenko et al., 2020). Interesting observations were made by the team of Oh et al. (2018), who showed that iron ions are a key factor stimulating the biofilm formation in *Campylobacter jejuni* by inducing oxidative stress in these bacteria (Oh et al., 2018). In this context, it is worth recalling the recently published results of Zhao et al. (2021), who in turn observed that NapA protein is expressed at a high level in the biofilm phase of *H. pylori* and contributes to the induction of the development of this biostructure by the oxidative stress stimulation (Zhao et al., 2021). We believe that the results obtained in this article perfectly complement the above-described observations, as they indicate that secretion of bOMVs by *H. pylori* may be a key mechanism involved in iron uptake and oxidative stress-dependent stimulation of biofilm development, as well as a buffering effect associated with numerous antioxidants protecting against the bactericidal action of iron ions. Although the relationship between the presence of iron ions in the environment and the formation of biofilm by *H. pylori* has so far been characterized only on the basis of microcolony formation on cell lines (Tan et al., 2009, 2011), we believe that our results may be a starting point for the development of research on anti-biofilm therapies against *H. pylori* aimed at iron uptake and metabolism. This type of concept has been extensively described in a review about pathogens from the ESKAPE group (Post et al., 2019).

The last group of proteins abundant in bOMVs from late growth phases of *H. pylori* constitute virulence factors associated with the promotion of carcinogenesis. Within these proteins, the presence of components involved in many different key steps in the *H. pylori* pathogenesis was noticed, including adhesion, local induction of immunosuppression and tissue destruction-related tumorigenesis promotion. The important enriched adhesins in *H. pylori* bOMVs were the well-known HP_1243 (BabA/OMP28) and HP_0896 (BabB/OMP19), both of which work synergistically in the recognition of mucins and Lewis antigens, leading to the establishment of effective gastric colonization (Kashani et al., 2008; Doohan et al., 2021). Importance of other two abundant adhesins, namely HP_0305 (YceI domain-containing protein) and HpaA (neuraminyllactose-binding hemagglutinin), was shown just recently (Fan et al., 2020; Varga et al., 2020). Interestingly, high hopes are currently placed in the use of HpaA in the production of vaccines against *H. pylori*, which is related to the high specificity and immunogenicity of this protein (Yang et al., 2019). HpaA is also known for its role in promoting adhesion (Carlsohn et al., 2006) which indirectly correlates to *H. pylori* carcinogenic potential. In fact, as demonstrated by Xia and colleagues in 2020, HpaA stimulates CD4^+^T cells to release IL-21 which induces an increased expression of metalloproteinases in AGS cells; this evidence supports the hypotesis that HpaA might play a significant role in the development of gastric carcinoma metastasis (Carlsohn et al., 2006). VacA, HP_1125 (OMP18, peptidoglycan-associated lipoprotein), HP_1177 (HopQ/OMP27) and HP_1286 (YceI domain-containing protein) are worth mentioning as enriched components of *H. pylori* bOMVs involved in promoting immunosuppression in the gastric environment. Overall, their activity contributes to the destruction of various subpopulations of immune cells (e.g., lymphocytes and macrophages) and the consequent reduction of the host’s antimicrobial activity against *H. pylori*, promoting long-term gastric colonization by this pathogen (Shan et al., 2015; Djekic and Müller, 2016; Tavares and Pathak, 2017; Gur et al., 2019; Behrens et al., 2020). The last discussed group of proteins located abundantly within bOMVs are components promoting tissue destruction and induction of gastric cancers. VacA and HP_1118 (*γ*-glutamyltranspeptidase) are together associated with disruption of the epithelial integrity by induction of both apoptosis and cell–cell junctions’ loosening (Ling et al., 2015; McClain et al., 2017; Bravo et al., 2019). Additionally, it was shown that HP_1118 has the ability to enhance a VacA-dependent vacuolation and induction of bacterial internalization into human gastric cells (Ling et al., 2015; Bravo et al., 2019). The detection of VacA suggests that bOMVs might contribute to cancer promotion given that this protein can trigger numerous disturbances that contribute to carcinogenesis such as induction of gastric epithelial cells death, pro-inflammatory activity and dysregulation of immune mechanisms, which reduces tumor surveillance (Carlsohn et al., 2006). Another two factors with synergistic action are HP_1456 (Lpp20) and HP0596 (tumor necrosis factor *α*-inducing protein), both being structural homologues that promote epithelial-mesenchymal transition and carcinogenesis (Godlewska et al., 2008; Vallese et al., 2017). It is worth mentioning that some of the adhesins, such as HP_0305 and BabA/BabB, may also directly or indirectly contribute to the induction of gastric inflammation (Varga et al., 2020; Doohan et al., 2021).

Summarizing, in the current original article it was observed that OMVs isolated from the late stationary phases of *H. pylori* biofilm, contain several important virulence factors that may be involved in tumor development. These findings are in line with the current scientific trend highlighting the importance of *H. pylori* biofilm in the progression of tumorigenesis (Rizzato et al., 2019). Indeed, biofilm formation increases the persistence and protection of the bacterium from immune responses as well as from the action of antibiotics contributing to the maintenance of a state of chronic inflammation in the gastric mucosa (Hathroubi et al., 2018; Choi et al., 2023). Furthermore, it has been shown that key virulence factors of *H. pylori,* including HP-NAP protein, indirectly promote biofilm formation and stability, further enhancing the processes that support gastric carcinogenesis (Liu et al., 2024). Collectively, while highlighting the need for more physiologically complex experimental models, this evidence indicates that biofilm and bOMVs do not represent simple survival mechanisms, but are capable of reshaping the tissue microenvironment towards conditions favorable to neoplastic transformation. Further studies are needed to evaluate the contribution of OMVs to carcinogenesis, starting from *in vitro* cell models where vesicles mechanism of action could be better investigated. Moreover, authors acknowledge that the *in vitro* model used to develop *H. pylori* biofilm in this study may have some limitations given that the vesicle composition is influenced by the growth conditions. Surely, the use of a more complex system, that can mimic the gastric environment, may help obtaining vesicles that better resemble those produced *in vivo.* However, the characterization of pOMVs and bOMVs performed in this study could be a starting point for the possible use of such structures as biomarkers for both the prevention of *H. pylori* infection and the development of vaccines.

Overall, these results provide a solid descriptive foundation for understanding how *H. pylori* biofilm and OMVs contribute to pathogenesis, while underlining the need to further clarify the molecular events highlighting their role in chronic infection and tumorigenesis. Future studies will be necessary to better understand the protein sorting and the biogenesis of OMVs associated with the biofilm phenotype. In particular, the construction of mutant strains for key genes such as the *tol-pal* gene cluster could be used to speculate the mechanisms of bOMVs biogenesis and vesicles content, as already demonstrated for the planktonic phenotype (Turner et al., 2015).

## Data Availability

The datasets generated and analyzed in the current study are available from the corresponding author on reasonable request. The mass spectrometry proteomics data have been deposited to the ProteomeXchange Consortium via the MASSivepartner repository with the dataset identifier MSV000098873 and PXD067502.

## Footnotes

1
https://www.uniprot.org/

2
http://bioinformatics.sdstate.edu/go/

3
https://string-db.org/
