# Supplementary material for: Characterization of Helicobacter pylori Outer Membrane Vesicles over time, in biofilm and planktonic phenotypes
Source: Front Microbiol. 2026 Apr 20;17:1765988. doi: 10.3389/fmicb.2026.1765988 (PMC13136172; doi:10.3389/fmicb.2026.1765988)
Supplement: Supplementary file 1 [file Data_Sheet_1.pdf]

Supplementary Material

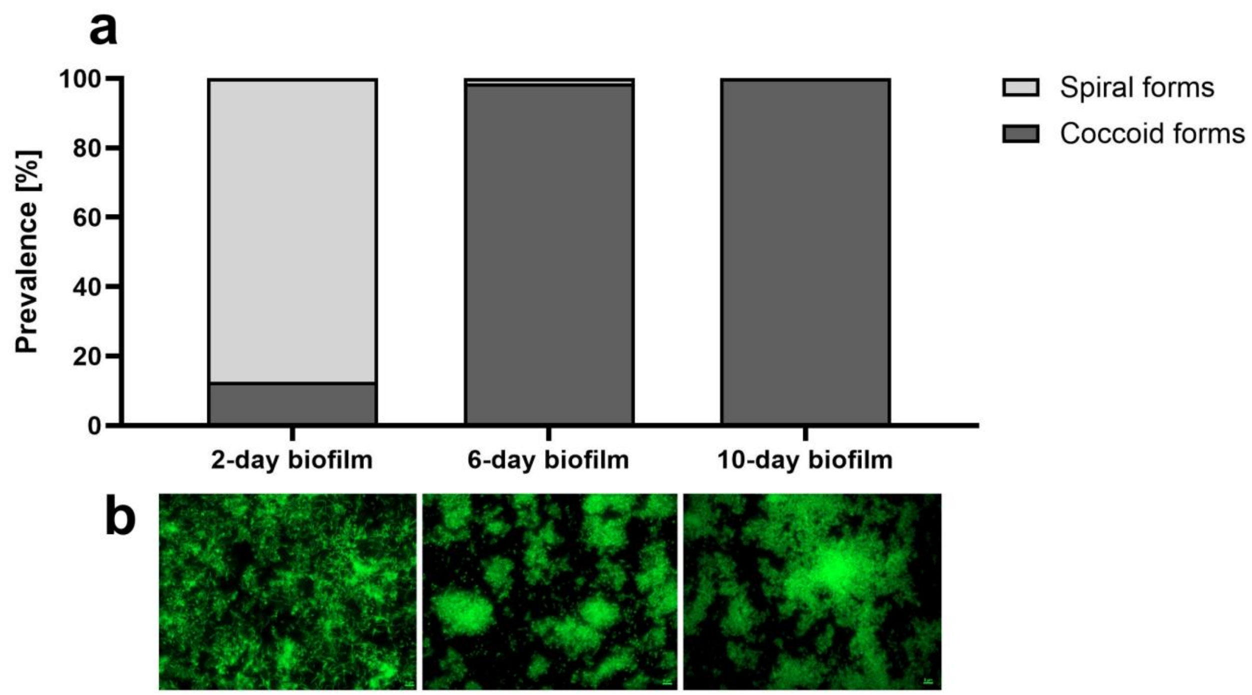

Supplementary Figure 1. Distribution cell morphology of *H. pylori* ATCC 43629 biofilms at different time points. Graph (a) and fluorescent images (b) demonstrating a time-dependent switch in spiral-to-coccoid morphology.

Supplementary Table 1. p-values determination from ANOVA.

	p-value		
**Samples**	**Vesicles/mL**	**Mean Diameter (nm)**	**Mode (nm)**
**Planktonic over time**	0.0006	0.0035	0.7564
**Biofilm over time**	0.0078	0.0011	0.0407

Supplementary Table 2. OMVs/bacterial cells ratios at each time point for planktonic and biofilm phenotypes.

Incubation Time	OMVs/planktonic cells ratio	OMVs/biofilm cells ratio
2 days	1.29×10^{5}	4.54×10^{1}
6 days	2.32×10^{9}	2.04×10^{3}
10 days	4.17×10^{6}	3.46×10^{3}

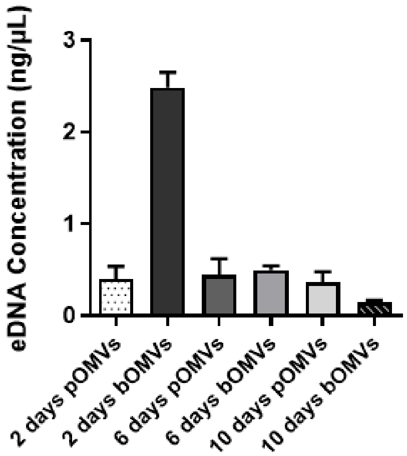

Supplementary Figure 2. eDNA concentration in *H. pylori* OMVs.
